# Meta-Narrative Review of Molecular Methods for Diagnosis and Monitoring of Multidrug-Resistant Tuberculosis Treatment in Adults

**DOI:** 10.4103/ijmy.ijmy_135_18

**Published:** 2018

**Authors:** Peter M. Mbelele, Sagal Y. Mohamed, Elingarami Sauli, Emmanuel A. Mpolya, Sayoki G. Mfinanga, Kennedy K. Addo, Scott K. Heysell, Stellah G. Mpagama

**Affiliations:** 1 Kibong’oto Infectious Diseases Hospital, Sanya Juu, Siha, Kilimanjaro; 2 Department of Global Health and Biomedical Sciences, School of Life Science and Bioengineering, Nelson Mandela-African Institution for Science and Technology, Arusha; 3 Division of Infectious Diseases and International Health, University of Virginia, Charlottesville, Virginia, USA; 4 Muhimbili Centre, National Institute for Medical Research, Dar es Salaam, Tanzania; 5 Department of Bacteriology, Noguchi Memorial Institute for Medical Research, University of Ghana, Accra, Ghana

**Keywords:** Anti-tuberculosis therapy, diagnosis, drug-resistant tuberculosis, molecular methods, monitoring

## Abstract

Early and accurate diagnosis and rigorous clinical and microbiological monitoring of multidrug-resistant tuberculosis (MDR-TB) treatment can curb morbidity and mortality. While others are still under evaluation, the World Health Organization has recommended few novel molecular methods for MDR-TB diagnosis only. We present current molecular methods for diagnosis and monitoring of MDR-TB treatment in TB-endemic settings. A systematic meta-narrative review was conducted according to the RAMESES recommendations. Electronic databases were searched for relevant articles published in English language from January 2013 to June 2018. Based on predefined criteria, two independent reviewers extracted the key messages from relevant articles. Disagreement between them was resolved through discussion and the involvement of a third reviewer, if needed. Key messages were synthesized to create the meta-narratives for method’s accuracy, drug-susceptibility capability, and laboratory infrastructure required. We included 33 articles out of 1213 records retrieved, of which 16 (48%) and 12 (36%) were conducted in high- and low-TB-endemic settings, respectively. Xpert® MTB/RIF, GenoType MTBDRplus, GenoType MTBDRsl, FlouroType™ MTBDR, TB TaqMan® array card, and DNA sequencers can accurately guide effective treatment regimens. Molecular bacterial load assay quantifies mycobactericidal impact of these regimens. Although they present inherent advantages compared to the current standard of care, they carry important limitations to implementation and/or scale-up. Therefore, considerable effort must now be directed to implementation and health systems research to maximize these forecasted benefits for individual patient’s health outcomes.

## Introduction

Treatment of multidrug-resistant tuberculosis (MDR-TB) defined as TB disease caused by *Mycobacterium tuberculosis* complex (MTBC) strains with resistance to rifampicin (RIF) and isoniazid is complex.^[1]^ There are now multiple treatment regimens and duration options for MDR-TB, depending on patient characteristics and MTBC drug-susceptibility testing (DST) results.^[1,2]^ These regimens contain at least five effective drugs consisting of one fluoroquinolone (FQ: levofloxacin, moxifloxacin, and gatifloxacin), one second-line injectable drug (SLID: kanamycin, amikacin, and capreomycin), two other core drugs (e.g., ethionamide/prothionamide, cycloserine/terizidone, linezolid, and clofazimine), and add-on drugs (high-dose isoniazid, pyrazinamide, and ethambutol). Pre-extensively drug-resistant TB (pre-XDRTB) and XDR-TB are defined as either resistance to FQs or SLIDs for pre-XDR TB or resistance to both FQs and SLIDs for XDR-TB. Both require individualized regimens by substituting the offending drugs, preferably with a drug such as bedaquiline.^[2,3]^ Evidence from South Africa TB program shows that bedaquiline-containing regimens for treating MDR-TB and XDR-TB patients considerably reduced mortality as compared to other regimens. The recent WHO rapid communication prefers an all oral bedaquiline-containing second-line regimen, should DST results otherwise allow.^[4]^ The new World Health Organization (WHO) guidance has not only emphasized the importance of DST but also generally ushered in a new era of more personalized focus on bedaquiline-containing treatment regimens.^[5]^ Regardless of regimen used, all patients with MDR-TB require routine monthly microbiological monitoring to track their progress and identify treatment failures or reversions.^[6,7]^

Despite advances in diagnosis and treatment of MDR-TB in the past decade, incomplete DST and inability to rigorously monitor microbiological response to anti-TB therapy with the current technologies make it difficult to effectively treat patients. Consequently, approximately 50% of the patients receiving MDR-TB treatment have unfavorable outcomes. In many TB-endemic settings, DST to all drugs in the regimen is not performed. This exposes patients to fewer active drugs, increasing their risk of acquiring drug-resistance, and undue toxicity if treated with a potentially harmful medication with little or no *in vivo* benefit.^[8,9]^ The End TB Strategy of the WHO aims to decrease TB incidence and mortality by 90% and 95%, respectively, by 2035. This will only be possible if a comprehensive series of interventions is utilized including field-tested methods for rapid diagnosis, more complete DST, and thorough monitoring anti-TB therapy.^[10,11]^ Until now, only Xpert® MTB/RIF (Cepheid, Sunnyvale, California, USA) and the line-probe assays GenoType® MTBDRplus and GenoType® MTBDRsI (both Hain LifeScience GmbH, Nehren, Germany) have received the WHO approval and all have important positive and negative performance characteristics and drug-susceptibility capabilities.^[12]^ There is no molecular method endorsed for monitoring treatment response at this time, necessitating the continued use of phenotypic methods.^[12,13]^ However, while serving as the gold standard for both DST and monitoring of microbiological response to treatment, phenotypic methods suffer practical limitations.^[13,14]^ First, the specimen must be processed to amplify the number of MTBC before DST and this must be performed in a Biosafety Level 3 Laboratory (BSL 3), which may not be easily found in a differentially resourced area. Furthermore, the results can be delayed for 4–12 weeks and up to 15% of the samples may be contaminated; this is a significant barrier for timely case management and can hinder infection prevention and control practices.^[15–17]^ In addition, routine culture misses more dormant nonreplicating MTBC subpopulations which can cause reversion to culture positivity after negative results or lead to relapse after therapy is completed.^[18,19]^

Most reviews have evaluated a single molecular method or compared the performance of multiple tests in TB-endemic, usually focusing on methods endorsed by the WHO using narrative approach.^[20,21]^ This approach does not give readers an opportunity to assess methodological process, and it suffers study selection bias.^[22]^ This review summarizes the current publicly available molecular methods for MDR-TB diagnosis and monitoring of treatment response using a systematic meta-narrative approach and focusing on advantages and limitations and concluding with an informed assessment of future directions for the field.

## Methods

### Study design and inclusion criteria

This systematic review was conducted within meta-narrative format, which qualitatively discusses a diverse concepts of molecular methods by highlighting the contrasting and complementary ways from different researchers.^[23]^ A protocol containing a set of eligibility criteria was developed and approved by authors according to the RAMESES meta-narrative review publication standards. Articles were included in the review if they met the following criteria: (i) original article published in English language from January 2013 to June 2018, (ii) cross-sectional or cohort studies that evaluated molecular method’s technical performance (sensitivity, specificity, and accuracy or concordance) for either MDR-TB diagnosis or monitoring anti-TB therapy using either sputa or isolates, and (iii) adult participants aged ≥18 years with presumptive pulmonary TB. Articles with the following features were excluded: (i) case reports; (ii) review articles, commentary articles, and short communications; (iii) epidemiological studies describing molecular epidemiology, drug resistance profile, case detection/notification rates, or lack of DST results; (iv) author evaluated multiple at once or an outmoded version of a method; (v) use in extra-pulmonary TB; and (vi) immunological or host biomarkers either for diagnosis of MDR-TB or monitoring anti-TB therapy.

### Search strategies and changes in the review process

In this review, recent evidence on molecular methods for monitoring anti-TB therapy was sparse. Therefore, the article search was extended to articles published from January 2011. We first searched Medline/PubMed, and then additional articles were obtained from Google Scholar and through scanning citations. Searching for relevant articles was conducted using the following terms: (molecular OR genotyp* OR “polymerase chain reaction” OR “PCR”) AND (“drug resistan* tuberculosis”) AND diagnosis OR (molecular or genotyp* OR “polymerase chain reaction” OR “PCR”) AND (“multidrug resistan* tuberculosis”) AND monitor* AND (“tuberculosis treatment response” OR “anti-tuberculosis therapy”).

### Selection and appraisal of articles

Two independent reviewers (PMM and SYM) screened the titles and abstracts of identified articles as per the eligibility criteria. An article was read in full if the abstract mentioned, in some capacity, performance of molecular method for DR-TB diagnosis or for monitoring anti-TB therapy. Duplicates were removed. A final consensus was discussed between the two reviewers. An opinion from a third reviewer (SGM) was sought for any disagreement between the two. Ultimately, eligible articles were archived in Mendeley-reference management Software (www.mendeley.com) referencing manager.

### Data extraction

A standardized data extraction form was developed, piloted, and revised to improve clarity. Independently, the two reviewers extracted data such as author’s name, year of publication, country, name of molecular method, target biomarker, intended use, study population, type and number of specimens tested, and the method’s technical performance measured against either phenotypic culture or genotypic-based DST from the relevant articles. To establish agreement between culture and molecular method in monitoring therapy, correlation coefficient and bacterial load decline rate were also extracted.

### Data analysis and synthesis

Characteristics of articles and molecular methods identified are summarized in Tables 1 and 2. Meta-narratives for different molecular methods from different articles were catalogued to illuminate the clinical applications and research opportunities in TB-endemic settings. They were featured to describe the principle of the test, technical performance (accuracy), advantages and limitations based on simplicity, turnaround time, laboratory infrastructure, and logistics required.

## Results

### Selection of studies included

**Figure 1:**
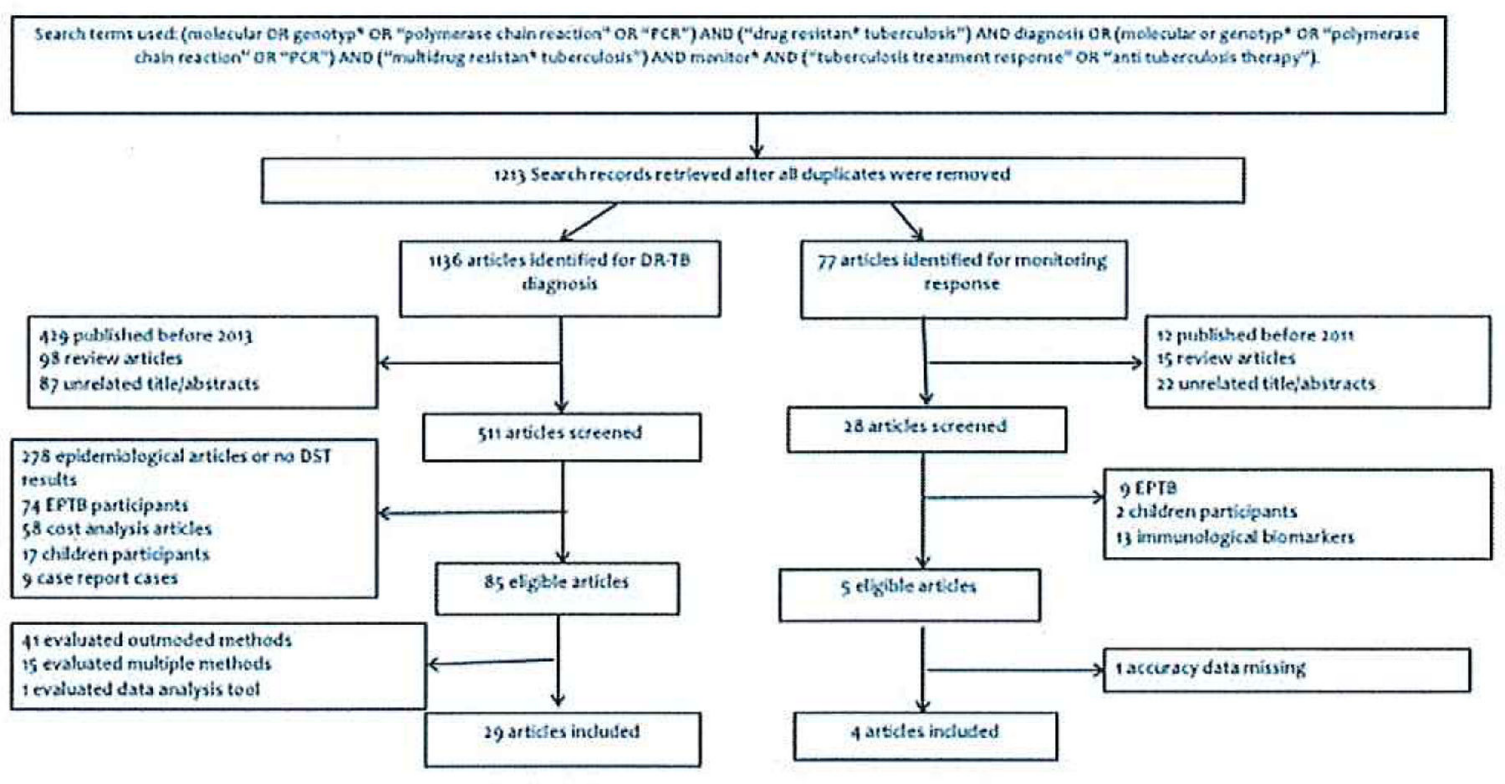
The processes and procedures used to retried relevant articles in electronic data. It also depicts the numbers of articles identified, screened for eligibility, and inclusion into the review

A total of 1213 articles were retrieved from all electronic databases. Of these, 92 articles (87 for diagnosis and 5 for monitoring therapy) were read in full. A total of 29 and four articles were included in the review of methods for diagnosis and monitoring anti-TB therapy, respectively [Figure 1], Reasons listed in Figure 1 are used to exclude irrelevant articles. Common molecular methods are summarized in Tables 1 and 2.

### Characteristics of articles included

This review included 33 articles published from 2011 to 2018 [Table 1 and Figure 1]. Of 33 articles, 16 (48%) were conducted in high TB-endemic settings, 12 (36%) in lower TB-endemic settings, and 5 (16%) had collaborators from both settings [Table 1]. About 64% (21/33) of articles reported methods that analyzed sputa. The target biomarkers, clinical application, and strengths and limitations of molecular methods are summarized in Table 1.

### Molecular methods for detecting *Mycobacterium tuberculosis* complex and multi/extensively drug-resistant tuberculosis

#### Xpert® MTB/RIF assay (Cepheid, Sunnyvale, California, USA)

The Xpert® MTB/RIF is a cartridge-based real-time polymerase chain reaction (RT-PCR) assay approved by the WHO for dual detection of MTBC and RIF susceptibility.^[24]^ It amplifies the target 560 region of MTBC and 81-bp RIF-resistant determining region (RRDR) in the codons 507–533 of the *rpoB* gene, a proxy biomarker for RIF-resistant TB (RR-TB).^[25]^ Xpert® MTB/RIF is robust and rapid, providing results within 24 h, and has sensitivity and specificity of over 95% and 99% in detecting RR-TB as compared to culture-based DST [Table 1].^[26,29]^ In addition, the test is simple to use and semi-automated with minimal risk of contamination and infection to laboratorians.^[30]^ However, susceptibility testing is limited to RIF only.^[24]^ It also requires laboratory infrastructure such as a stable electrical supply and a consistent temperature-humidity range necessary to prevent module malfunctions.^[31]^

#### Xpert® MTB/RIF Ultra assay (Cepheid, Sunnyvale, California, USA)

Xpert® MTB/RIF Ultra assay (Ultra) is a new generation assay that is more sensitive than Xpert® MTB/RIF (Cepheid, Sunnyvale, California, USA). The Ultra detects two additional multi-copy amplification targets (IS6110 and IS 1081) and has a larger PCR chamber to accommodate 50 µL of a sample compared with 25 µL in Xpert® MTB/RIF. This design lowers the limit of detection from 131 CFU/mL for Xpert® MTB/RIF to 16–20 CFU/mL, accounting for sensitivity of 93% for Ultra compared to culture [Table 1].^[32,31]^ It detects MTBC even in patients with paucibacillary load. Unlike Xpert® MTB/RIF, Ultra uses melting temperatures instead of the RT-PCR curve analysis, which allows detection of silent mutations within RRDR that may or may not be associated with resistance. Therefore, Ultra is robust and has improved ability to detect mutations predictive of phenotypic RIF resistance (i.e., *rpoB* 533 C-to-G mutations), while avoiding false positives in samples with low bacterial load [Table 1].

#### Xpert XDR assay (Cepheid, Sunnyvale, California, USA)

The XDR assay, also called Xtend XDR, is a new CEPHEID platform for detecting pre-XDR and XDR-TB. Principally, the assay is designed into three phases: 8-plex nested PCR, melt curve analysis, and 10 sloppy beacon detection probes. The assay can differentiate 32 mutations in genes predictive of phenotypic resistance to isoniazid (*katG* and *inhA* promoter genes), FQs (*gyrA* and *gyrB*), and aminoglycosides *(rrs* and *eis* promoter). Compared to sequencing, XDR assay has sensitivity of 98%, 96%, 93%, and 97% in detecting isoniazid, FQ, and aminoglycoside (kanamycin and amikacin) resistance, respectively, with specificity of 100%. The sensitivity is lower when compared with Conventional MGIT 960 System at 71%, 83%, and 88% in detecting aminoglycoside, isoniazid, and FQ resistance, respectively [Table 1].^[34,35]^ Unfortunately, this assay has not yet been evaluated in clinical settings or in implementation studies. The XDR assay also cannot reliably predict susceptibility for the cyclic polypeptide capreomycin, an alternative drug to the aminoglycosides.

#### GenoType® MTBDR assays (Hain Lifescience GmbH, Nehren, Germany)

The GenoType® MTBDR is DNA-strip line probe assay (LPA) that amplifies MTBC DNA and drug RDRs and detects mutation(s) on target genes predictive of MDR-TB or XDR-TB. For example, the genotype MTBDRplus version 2.0 dually detects MTBC and mutations predictive of phenotypic resistant to RIF on *rpoB* and isoniazid on both *katG* and *inhA* genes.^[36]^ Compared to culture-based DST, it has sensitivity and specificity of 84% and 98%, with accuracy of 83% in detecting MDR-TB [Table 1].^[37–45]^ This performance is also similar for genotype MTBDRsl version 2.0 in detecting mutations on *rrs and eis* promoter regions, predictive of phenotypic resistance to amikacin and kanamycin, and *gyrA and gyrB* genes for FQs [Table 1].^[46–48]^ Compared to the Xpert® MTB/RIF platforms, the GenoType MTBDR assays are more labor intensive. They require a skilled laboratorian, adequate laboratory infrastructure compatible with at least BSL2, biosafety cabinets, three separate rooms to accommodate all steps and minimize cross-contamination risk, constant power supply, refrigerator or freezer to store reagents and centrifuges.^[36,49]^

#### FluoroType® MTBDR assays (Hain Lifescience GmbH, Nehren, Germany)

The FluoroType® MTBDR assay is a semi-automated LPA that detects MTBC DNA and mutations on *rpoB* for RIF and both *katG* and *inhA* genes for isoniazid from both isolates and sputum samples.^[50,51]^ This detection is made in a closed system using melting-curve analysis and the results are read and provided by FluoroSoftware in 3–4 h.^[50,51]^ Compared to phenotypic DST, sensitivity in detecting RIF and isoniazid resistance is 99% and 92%, respectively, with specificity of 100%.^[50]^ In sputum samples, the assay has excellent sensitivity of 100% and specificity of 97%, compared to GenoType® MTBDRplus or targeted Sanger sequencing.^[51]^ Its main advantage over other LPAs is its closed system and automation that reduces the risk of contamination and erroneous results interpretation. Like other LPAs, it requires different workstations for DNA extraction and preparation of PCR mix and hybridization.

#### TaqMan® array card for tuberculosis

The TaqMan® array card for TB (TB-TAC) is a customizable 384-well microfluidic RT-PCR system that compartmentalizes each sample into 48 different PCRs simultaneously for detecting mutations on multiple genes associated with phenotypic resistance of MTBC to anti-TB drugs.^[52]^ These genes include *inhA* and *katG* (isoniazid), *rpoB* (RIF), *embB* (ethambutol), *rrs* (kanamycin, amikacin, and capreomycin), *eis* (low-level kanamycin), *gyrA* and *gyrB* (FQs), 23S and *rplC* (linezolid), and *pncA* (pyrazinamide). TB-TAC has two layers of detection: the probe-based layer, containing over 40 sequence-specific probes, and the second layer, high-resolution melt (FIRM) analysis interrogated into at least 20 primer pairs and 27 amplicons for detecting MTBC and the presence of wild-type and mutant genes encoding these drugs. It also characterizes *pncA* mutations which are not possible with probe-based assays. The assay performs more accurately in smear-positive sputum than smear-negative samples at 89% and 33%, respectively, as compared to culture and Sanger sequencing. The overall accuracy for MTBC susceptibility to all anti-TB drugs is 87% [Table 1].^[52–54]^ However, it requires an expensive RT-PCR platform and skilled personnel to interpret FIRM software results and has only been used in the research settings.

#### DNA sequencing technologies

DNA sequencing technologies have gained popularity, not only in research settings but also in clinical applications and public health and epidemiological investigations.^[55]^ Principally, all sequencing technologies involve DNA extraction, library preparation by breaking down genomic DNA into small base paired fragments, sequencing to 100–300 bp reads, and ultimately curating of sequence reads. Adequate quality reads are then mapped to published M *tuberculosis* reference genome sequences to identify single nucleotide polymorphisms and insertions-deletions.^[55]^ Finally, bioinformatic analyses are carried out to interpret results and predict strain lineage and drug resistance using different software tools.^[56]^ Sequencing is accurate, robust, and average turnaround time is 7 days [Table 1]. For example, whole genome sequencing (WGS) by Illumina MiSeq platform can differentiate MTBC species, detect, and predict ding resistance phenotypes at a sensitivity of 99%, 96%, and 83%, respectively. Compared to either culture or genotypic-based DST, the concordance, sensitivity, and specificity of WGS in detecting phenotypic resistance to anti-TB drugs range from 83% to 99%, 83% to 100%, and 78% to 99%, respectively [Table 1].^[57–60]^ Sequencing allows tracing of genetic relatedness and transmission dynamics of MTBC strains during an outbreak.^[55]^ It is expensive, mostly done in reference research or clinical laboratories by skilled bioinformaticians and requires several software for analysis, stable internet access, and a regularly maintained hardware server for online storage of biological data. In addition, sequencing has no standardized protocol or testing algorithms across the globe. Nevertheless, as the technology moves closer to point of care, there will be numerous opportunities for implementation studies.

### Molecular methods for monitoring anti-tuberculosis treatment

#### Xpert® MTB/RIF assay and propidium monoazide

DNA-based molecular methods such as Xpert and LPA are not recommended for monitoring treatment response in patients with tuberculosis because they cannot differentiate viable and dead MTBC DNA.^[ 25]^ However, pretreatment of sputum samples with propidium monoazide (PMA) (Biotium Inc., Hayward, California, USA) increases the specificity of Xpert® MTB/RIF in the detection of viable DNA. PMA selectively intercalates the dead MTBC DNA and inhibits its amplification and detection.^[61]^ Monitoring anti-TB treatment by Xpert-PMA has been evaluated in two studies. The first study measured bacterial load from 1937 sputum samples that were collected before treatment, 2 weeks after treatment, and monthly thereafter, during the intensive and continuation phases of non-MDR-TB and MDR-TB treatment.^[62]^ In the second study, participants produced 151 sputa at eight time points before treatment and then at days 3, 7, 14, 28, 35, 56, and 84 of treatment.^[63]^ Compared to culture, both studies achieved 53%−80% specificity for detecting viable MTBC DNA [Table 1].

#### Molecular bacterial load assay

Molecular bacterial load assay (MBLA) is a RT-PCR that detects and quantifies 16S ribosomal RNA (16S rRNA) of viable MTBC from sputa.^[64]^ When MTBC cells are killed by anti-TB drugs, the amount of rRNA also decreases, making it possible to estimate the number of viable cells in sputum sample. rRNA decline has been interpreted as a surrogate biomarker of bactericidal activity for anti-TB therapy. For instance, two studies documented mean MTBC load decline rate and correlation of MBLA with culture from sequential sputum samples for at least 14 days of intensive phase of treatment in patients treated for drug-susceptible TB of 90% and 84%, respectively [Table 1].^[64,65]^ MBLA is rapid, robust, and accurate with minimal or no risk of contamination [Table 1]. Nevertheless, logistics for handling sputum samples have not yet been optimized for use in clinical settings. It is also expensive and requires skilled personnel for several manual steps and good laboratory infrastructure compatible with reference-level laboratories.

## Discussion

In this meta-narrative review, we report rapid and accurate molecular methods for MDR-TB diagnosis and monitoring anti-TB therapy. Their rapidity shorten the time to diagnosis and treatment from 2 to 3 months by phenotypic culture to 1–7 days.^[66]^ They accurately guides early treatment options that both minimize transmission and further development of drug resistant strains in the community.^[67]^ Although implementation of molecular methods for diagnosis reduced time to treatment of MDR-TB in South Africa and Georgia,^[68,69]^ high cost and unavailability of comprehensive implementation and impact assessment plans in most TB-endemic settings have been the main constraints to incorporate in clinical practices.^[69,70]^ Furthermore, a cluster-randomized clinical trial in South Africa found that usage of Xpert® MTB/RIF in initial TB diagnosis did not provide a mortality benefit as compared to smear microscopy.^[71]^ Further evaluation of these diagnostics especially those with expanded DST in clinical settings is required to improve patient care and reduce global TB burden.

Multiple molecular methods such as TAC-HRM and DNA sequencing technologies, which extend DST to most anti-TB drugs including pyrazinamide, have been appraised in this review.^[57,60]^ Unlike TAC-HRM and other probe-based assays, sequencing technologies can categorize mutations into high, moderate, or low confidence resistance patterns that may or may not be associated with phenotypic drug resistance. Technological advances from Sanger sequencing to next-generation sequencing (NGS) enhance detection of heteroresistance that can occur at very low levels within a specimen. In a multi-country study, amplicon-based NGS detected over 5% and 21% heteroresistant strains that were deemed wild-type and mutant by Sanger sequencing.^[72]^ While expanding the scope of anti-TB DST adds clinical value, these strains may have relevance in informing drug-susceptibility for antibiotics such as pyrazinamide and FQs and can contribute to the decision to initiate patients on a bedaquiline-based regimen and/or one supplemented by other drug classes.^[4]^ Generally, probe-based assays (with or without HRM) and sequencing methods have diagnostic value but have not been widely used in designing DR-TB treatment regimens. Unlike sequencing, which has a wider reach, probe-based methods target specific RDRs of a gene such as *vpoB* for RIF. This explains why some methods have limited DST capability. Noting that not all mutations lead to phenotypic resistance, their accuracy, and clinical impact requires parallel testing with conventional phenotypic DST methods.^[73]^

Even if the treatment regimen is well design for individual patient, regular microbiological monitoring, a key clinical practice to foresee health outcomes, is required throughout the duration of anti-TB therapy. Despite challenges related to smear microscopy for acid-fast bacilli and isolation of MTBC on culture, these methods remain the worldwide gold standard for monitoring anti-TB therapy. In this review, two applications of molecular methods used for monitoring anti-TB therapy have been highlighted. In the first method, serially measured MTBC 16S rRNA, a proxy biomarker for viability that assesses mycobactericidal decline during anti-TB treatment by MBLA, was compared to phenotypic culture-based methods.^[74]^ MBLA has not been recommended by the WHO, but its potential clinical value was reported in one case report that documented favorable outcomes after MBLA results were used to modify the anti-TB regimen. In this case, a 12-year-old child with TB/HIV coinfection had prolonged smear positivity beyond 2 months of standard treatment for drug-susceptible TB. MBLA showed high bacterial load in the 1^st^ month. After substitution of moxifloxacin for rifabutin, mycobacterial decline by MBLA was demonstrated, and culture was negative for the duration of treatment.^[75]^ In the second method, PCR inhibitors such as PMA (which bind the DNA of dead bacilli and allow for serial measurement of viable DNA) were used in conjunction with Xpert® MTB/RIF. However, specificity in detecting viable mycobacteria was low favoring serial measurement of 16S rRNA [Table 1]. Thus, there remains no molecular method recommended by the WHO to replace or complement phenotypic methods for monitoring anti-TB therapy.^[12]^ Yet, this meta-narrative review favors further clinical and implementation studies of both Xpert/PMA assay and MBLA to evaluate applicability in different settings and relevant patient outcomes.

This review also found unanswered questions on molecular methods necessary to guide choices for MDR-TB regimen, which merit further attention.^[76,77]^ Possibilities include further studies of TAC on direct sputum along with additional biomarkers that would improve detection in smear-negative individuals. Similarly, it is worth evaluating the Xpert-XDR assay and optimizing protocols for DNA extraction, sequencing, bioinformatics tools, and analysis to augment or replace conventional DST methods in patients who are currently being treated for DR-TB or are failing their regimens. Unlike drug-susceptible TB, treatment failure and relapse are more common in MDR-TB, and patients with pre-XDR and XDR-TB remain culture positive for long periods. These are resource-intensive conditions that require tests such MBLA for monitoring mycobactericidal activity and altering regimens for patients who are poorly responding to anti-TB therapy. In this review, 36% of the molecular methods were performed in lower TB-endemic settings. Because mortality is higher in high TB-endemic settings in comparison to low TB-endemic settings, this meta-narrative review supports recommendations that these settings require more investment and should lead the TB research agenda, a key step toward achieving the 2035 World End-TB strategy.^[78,79]^

### Strength and limitations

The main strength of this review is that it provides timely and relevant information on various molecular methods for diagnosis of MDR-TB and monitoring anti-TB therapy, and it explains potential clinical impact and research opportunities. Focusing on articles published in English language only may have limited the scope of information presented. However, previous systematic reviews documented that language restriction has no significant effect.^[80,81]^

## Conclusion

We found potential molecular diagnostic methods for MDR-TB diagnosis, expanded DST to tailor individualized regimens, and monitoring of treatment response. We urge funders to support efforts to evaluate these technologies for their various clinical applications. Meticulous introduction of these technologies in differing clinical settings will likely be a major step toward fulfilling the End TB Strategy.

## Figures and Tables

**Table 1:** Characteristics of articles and molecular methods evaluated in adults with multidrug resistance tuberculosis

Author and year	Country	Purpose	Target biomarker	Specimen tested	Sample size	Performance
Rice *et al*., 2017^[26]^	USA	Detects MTBC and	A 560 region of MTBC DNA and 81-bp of RRDR in the codons 507–533 of the *rpoB* gene	Sputum	751	100% sensitivity and 98% specificity
Guenaoui *et al*., 2016^[27]^	Algeria	DST of RIF		Sputum	50	Sensitivity and specificity arc 100%
Chikaonda *et al*., 2017^[28]^	Malawi			Sputum	348	Sensitivity and specificity of 100%
Huang *et al*., 2018^[29]^	China				1062	97% sensitivity and 98% specificity
Chakravorty *et al*., 2017^[32]^	USA		*rpoB* gene, IS6110 and IS 1081	Sputum	277	93% sensitivity and 98% specificity
Dorman *et al*., 2018^[33]^	South Africa, Uganda, Kenya, India, China, Georgia, Belarus, and Brazil			Sputum	314	95% sensitivity and 98% specificity
Chakravorty *et al*., 2017^[34]^	USA	Detects MTBC and DST of FQs and SLIDs (AMK and KAN), and INH	*gyrA, gyrB, katG.* and *rrs*	Sputum	24	100% sensitivity for all targets and >94% specificity compared to sanger sequencing. Using phenotypic DST, Sensitivity for FQ is 75% and 100% for INH and AMK, KAN. Specificity 100% for INH and FQ and 94% for SLIDS
Xie *et al*., 2017^[35]^	China and South Korea			MTBC isolates	308	Using culture/genotypic DST, sensitivity for INH, FQs and SLIDs is 83/96%, 88/98% and 71/93% respectively. Specificity was 94/99%
Chen *et al*., 2014^[37]^	China	Detects MTBC and DST of RIF and INH, the MDR-TB defining anti-TB agents	*rpoB. katG, inhA* genes	Sputum	427	86% sensitivity and 96% specificity for RIF; and 77 and 95% for INH. 70% sensitivity and 97% specificity for detecting MDR-TB and >70% accuracy against culture
Karimi *et al*., 2018^[38]^	Morocco			Sputum	70	92 and 97% sensitivity for RIF and INH respectively with 100% Specificity
Lin *et al*., 2017^[39]^	Taiwan			Sputum	5838	Sensitivity and specificity for RIF were 92 and 97% and 78% and 100% for INH. 83% sensitivity and 100% specificity for detecting MDR-TB, and >95% test accuracy
Maharjan *et al*., 2017^[40]^	South Africa			Sputum	69	Sensitivity and specificity for RIF, INH and MDR-TB were 89% and 100%
Maningi *et al*., 2017^[41]^	Nepal			MTBC Isolates	100	Sensitivity for detecting RIF and INH and MDR were 100%. Specificity was 88%, 94% and 100% respectively, and >70% accuracy with culture
Seifert *et al*., 2016^[42]^	USA, India, Moldova, and South Africa			Sputum	1128	Sensitivity and specificity for RIF were 97% and 98% and 94% and 100% for INH. 95% sensitivity and 99% specificity for detecting MDR-TB
Abanda *et al*., 2017^[43]^	Cameroon			Sputum	225	Sensitivity for detecting RR, INH, and MDR-TB 98%, 92$, 94% respectively, and specificity over 99% with 96% accuracy with culture
Maeza *et al*., 2017^[44]^	Ethiopia			Sputum	274	sensitivity for detecting RR, INH and MDR-TB 88, 92, 96% respectively, and specificity over 99%
Singh *et al*., 2017^[45]^	India			Sputum	572	Sensitivity for RIF and INH were 100 and 99%, with 99% specificity
Tagliani *et al*., 2015^[46]^	Europe (Germany, Italy and Sweden)	Detect MTBC and DST to SLIDs and FQs (either XDR or pre-XDR-TB)	*rrs, eis, gyrA* and *gyrB* on the DNA of MTBC	MTBC Isolates	228	86% and 90% Sensitivity and specificity for SLIDs, 83%−94$ and 100% for FQs, and 80$ and 82% for detecting MDR-TB, respectively
				Sputum	231	Sensitivity and specificity for SLIDs were 90% and 92%, and 93$ and 98% for FQs and for detecting MDR-TB was 82% and 98% respectively
Gardee *et al,* 2017^[47]^	South Africa			MTBC Isolates	268	89% and 99% sensitivity and specificity for SLIDs and 100% and 99% for FQs, and 87% sensitivity for detecting XDR-TB, with 96% test accuracy for both target drugs
Yadav *et al*., 2018^[48]^	India			Sputum and isolates	431	93% sensitivity and 100% specificity for both SLIDs and XDR-TB, and 97% and 99% for FQs respectively
Hillemann *et al*., 2018^[50]^	Germany	Detects MTBC and DST of RIF and INH	*rpoB, katG, inhA* genes	Isolates	180	99% and 92% sensitivity for RIF and INH, respectively with 100% specificity compared to phenotypic DST
de Vos *et al*., 2018^[51]^	South Africa			Sputum	448	100% sensitivity for both RIF and INH is 100% with 97% and 98% specificity for RIF and INH respectively, compared to genotypic DST
Pholwat *et al*., 2015^[52]^	Bangladeshi, Thailand and Tanzania	Detects MTBC and DST for both first- and second-line drugs including PZA	*rpoB. katG. inhA, pncA. emb, rrs*, *eis. tlyA, rpIC, gyrA, gyrB etc*.)	MTBC Isolates	230	87% and 96% accuracy against culture and Sanger sequencing respectively. Accuracy for detecting PZA 81%
Foongladda *et al*., 2016^[53]^	Bangladeshi, Thailand and Tanzania			MTBC Isolates	212	75%−87% sensitivity and 91 %−98% specificity for SLD and≥91% for detecting MDR-TB with 87% accuracy for all drugs tested
Banu *et al*., 2017^[54]^	Bangladeshi and Thailand			sputum and MTBC Isolates	71	98% sensitivity and 92% specificity. Sensitivity in smear positive and negative was 89% and 33%, with 96%accuracy against Sanger sequencing
Walker *et al*., 2015^[57]^	UK	Detection of MTBC up to species level and associated phenotypic drug resistance	SNP on entire MTBC genome or target region of a gene	MTBC isolate	2099	92% sensitivity, 98% specificity, and 89% accuracy or detecting resistance
Quan *et al*., 2018^[58]^	UK			MTBC isolates	2039	94% sensitivity, 99% specificity, and 99% accuracy for detecting resistance
Chatterjee *et al*., 2017^[59]^	India			MTBC isolates	74	100% sensitivity, 94% specificity, and 97% accuracy for detecting MDR-TB
Shea *et al*., 2017^[60]^	New York State, USA			MTBC isolates	608	Sensitivity for speciation, detecting and predicting resistance for all drugs was 99, 96% and 83% respectively. Specificity and accuracy were 99%
Nikolayevskyy *et al*., 2015^[62]^	UK, Italy, Russia, Lithuania, Latvia	Detect viable MTBC DNA during treatment	PMA free DNA (viable DNA)	Serial sputa	1937	Sensitivity and specificity for detecting viable MTBC DNA was 98% and 71%−80%
Kayigire *et al*., 2016^[63]^	South Africa				151	Sensitivity and specificity for detecting viable MTBC DNA was 95% and 63% compared to 95% and 42% in samples without PMA respectively
Honeyborne *et al*., 2011^[64]^	Tanzania and Germany	Monitoring treatment response	MTBC 16S rRNA	Serial sputa	148	Biphasic decline of bacterial load in response to anti-TB treatment comparable o culture
Honeybome *et al*., 2014^[65]^	Tanzania				111	Biphasic decline as observed longitudinally during anti-TB therapy at a mean of 0.99–0.81 log

MTBC: Mycobacterium tuberculosis complex, MDR-TB: Multidrug-resistant tuberculosis, PZA: Pyrazinamide, PMA: Propidium monoazide, DST: Drug susceptibility testing, SLIDs: Second-line injectable drug, rRNA: Ribosomal RNA, SNP: Single nucleotide polymorphism, RIF: Rifampicin, INH: Isoniazid, XDR-TB: Extensively drug-resistant tuberculosis, AMK: Amikacin, KAN: Kanamycin, RRDR: Rifampicin resistant determining region

**Table 2:** Summary of molecular methods for either diagnosis or monitoring of drug resistant tuberculosis patients

Variable & methods	Xpert® MTB/RIF	Xpert®- Ultra	Xpert®- XDR	Genotype MTBDRplus	Genotype MTBDRsl	FlouroType MTBDR	TAC-HRM	DNA Sequencers	Xpert-PMA	MBLA
Maker	Cepheid, USA			Hain Life Science, Germany			Thermal fisher, USA	Several	Cepheid, USA	UK
WHO status	Approved		Not approved	Approved		Not approved				
Purpose	DR-TB diagnosis								monitoring therapy	
Target genes or biomarker	rpoB	rpoB, IS6110& IS 1081	kalG, inhA, gyrA, gyrB, rrs	rpoB, katG & inhA	gyrA, gyrB, rrs & eis	rpoB,katG & inhA	rpoB, katG, inhA, gyrA, gyrB, rrs, pncA etc.	All or several target genes	DNA for viable MTBC	I6S rRNA for viable MTBC
Anli-TB drugs	RIF	RIF	INH, FQs, KAN, AMK	RIF & INH	FQs, KAN & AMK	RIF & INH	Several e.g., PZA	All or target	Not applicable	Not applicable
TAT (days)	1	1	1	2	2	1	2	5–10	1	2
Specimen	Sputum & isolates	Sputum & isolates	Sputum & isolates	Sputum & isolates	Sputum & isolates	Sputum & isolates	Sputum & isolates	Isolates	sputum	sputum
Minimum Location	Peripheral laboratory			reginal and reference laboratory				reference laboratory	Peripheral laboratory	reginal laboratory
Infra structurer	BSL2 with BSC	BSL2 with BSC	BSL2 with BSC	BSL2 with BSC	BSL2 with BSC	BSL2 with BSC	BSL2 with BSC	BSL3	BSL2 with BSC	BSL3
Personnel required	less skilled laboratorian			Skilled laboratorian with molecular biology knowledge				highly Skilled	less skilled	skilled
Reagents storage	rT			2–8 °C and freezer (either −20 or-80 °C)					rT	2–8 °C and freezer
Reference	[26–29]	[32,33]	[34,35]	[37–45]	[46–48]	[50,51]	[52–54]	[57–60]	[62,63]	[64,65]

AMK: amikacin; BSC: biosafety cabinet; BSL: biosafety laboratory level; FQs: fluoroquinolones; INH: Isoniazid; KAN: Kanamycin; MBLA: Molecular bacterial load assay; PZA: Pyrazinamide; rT: room Temperature; TAT: turnaround time and TAC-HRM: TaqMan® array card-high resolution melts
